# Controlled Field Trial of Pancreas Disease Vaccines in Farmed Atlantic Salmon: Effects on Growth and Mortality During a SAV2 Outbreak

**DOI:** 10.1111/jfd.70003

**Published:** 2025-06-20

**Authors:** Jostein Mulder Pettersen, Marte Follesø Sønnervik, Marius Karlsen, Hege Jørstad Sekkenes, Geir Schriwer, Petter Gjesdal, Ane Sandtrø, Magnus Vikan Røsæg

**Affiliations:** ^1^ Salmalytics AS Oslo Norway; ^2^ PHARMAQ, Part of Zoetis Oslo Norway; ^3^ SalMar Farming AS Trondheim Norway

**Keywords:** Atlantic salmon, controlled field trial, pancreas disease, salmonid alphavirus subtype 2 (SAV2), vaccine effect

## Abstract

Pancreas disease (PD), caused by salmonid alphavirus (SAV), is a concern for salmon aquaculture in Scotland, Ireland and Norway. This article presents results from a controlled field trial monitoring three study groups from vaccination to harvest. The groups represented a subset of the total fish population within a single sea cage, reared under commercial farming conditions. The trial aimed to evaluate the effects of two licensed PD vaccines in Norway—ALPHA JECT micro 1 PD (AJm1PD) and Clynav—on growth and mortality during a PD outbreak, relative to an unvaccinated control group. Clinical PD, caused by SAV subtype 2, was confirmed in June 2024, approximately 14 months after sea transfer and 2 months before harvest. At harvest, both vaccinated groups exhibited significantly higher weights compared to the control group, with mean increases of 0.19 kg (95% CI: 0.05–0.33) for AJm1PD and 0.38 kg (95% CI: 0.24–0.51) for Clynav. Significant risk differences (%) in mortality were observed between the PD‐vaccinated groups and the control during the outbreak, with lower mortality in vaccinated fish (AJm1PD: −0.60 [95% CI: −0.98 to −0.22]; Clynav: −1.10 [95% CI: −1.45 to −0.74]). To account for potential misclassification of mortalities, bias‐adjusted estimates were calculated, revealing greater risk differences compared to unadjusted estimates (AJm1PD: −0.99 [95% CI: −1.53 to −0.45]; Clynav: −2.07 [95% CI: −2.58 to −1.56]). These findings suggest that failing to adjust for misclassification biases the results toward the null, leading to an underestimation of the true effect size. In contrast, risk ratios remained largely unaffected by these adjustments. Overall, the study suggests that both vaccines improved outcomes, with the DNA vaccine (Clynav) showing a more pronounced effect on both growth and survival compared to the inactivated SAV vaccine (AJm1PD), while also highlighting methodological challenges in conducting controlled field trials.

## 1 Introduction

Pancreas disease (PD) is an important disease in Scottish, Irish and Norwegian salmon aquaculture (Jansen et al. 2017). The disease is caused by a spherical, enveloped, single‐stranded RNA virus belonging to the Togaviridae family (Powers et al. 2001; Weston et al. 1999). While formally designated as salmon pancreas disease virus (SPDV), it is commonly referred to as salmonid alphavirus (SAV) (Weston et al. 2002). Seven subtypes (SAV 1–7) have been described (Fringuelli et al. 2008; Tighe et al. 2020). The predominant subtypes in Norway are SAV2 and SAV3, occurring in two separate regions: SAV3 is endemic from Hustadvika in mid‐Norway and southwards, while SAV2 is endemic from mid‐Norway and northwards up to the border of Trøndelag (Hjortaas et al. 2013, 2016; Jansen et al. 2017; Sommerset et al. 2024). Studies have reported differences in virulence between SAV subtypes, with SAV3 being more virulent than SAV2 (Graham et al. 2011; Jansen et al. 2015; Taksdal et al. 2015). However, the subtypes are serologically similar and induce cross‐immunisation, suggesting that a vaccine based on one subtype can provide protection against infection with a different subtype (Graham et al. 2014).

The disease occurs in farmed salmon during the seawater phase of production and can spread horizontally in water between populations (Aldrin et al. 2010; Kristoffersen et al. 2009; Stene et al. 2014; Viljugrein et al. 2010). Diseased salmon develop acute necrosis and loss of exocrine pancreatic tissue, myocardial necrosis with associated inflammation and degeneration and inflammation and degeneration of skeletal muscle (McLoughlin and Graham 2007). Outbreaks in salmon populations are characterised by appetite drops, altered behaviour, reduced growth and feed conversion ratio and increased mortality (Graham et al. 2010; Jansen et al. 2015, 2017; Jensen et al. 2012; Rodger and Mitchell 2007; Røsæg, Garseth, et al. 2019). In addition, the disease has been associated with filet quality problems and downgrading at slaughter (Larsson et al. 2012; Lerfall et al. 2012; Taksdal et al. 2012). Although the severity of the disease can vary substantially (Jansen et al. 2017), it can lead to significant economic losses to the fish farmer (Aunsmo et al. 2010; Pettersen et al. 2015). PD is a notifiable disease in Norway. According to current regulations, a formal diagnosis requires positive results from two independent diagnostic methods, such as PCR and histology (Anonymous 2017). In current diagnostic practice, these methods are applied to the same individual fish to confirm the presence of characteristic histopathological changes and the virus (Sommerset et al. 2024).

Vaccination, functional feed, improved genetics and biosecurity measures, including stamping out procedures, are applied for reducing losses and hindering transmission between farms (Jansen et al. 2017). A monovalent oil‐based PD vaccine containing inactivated SAV1 was first introduced to the market in 2007 (Norvax Compact PD; originally by Intervet International B.V, renamed MSD Animal Health in 2011). A multivalent oil‐based vaccine containing a PD component based on inactivated SAV1 became available in Norway from 2015 (Aquavac PD 7 vet.) but has later been withdrawn from the Norwegian market. Currently, two PD vaccines are licensed in Norway: a monovalent oil‐based PD vaccine containing inactivated SAV3 (ALPHA JECT micro PD1; PHARMAQ part of Zoetis), approved in 2015, and a DNA vaccine encoding structural proteins of SAV2 (Clynav; originally by Elanco Animal Health, owned by MSD Animal Health as of 2024), approved in 2017.

Documenting the effects and side effects of preventive measures, such as vaccines, is important for informed decision‐making and optimising resource allocation in disease management. Although several studies have demonstrated the effectiveness of PD vaccines under controlled laboratory conditions (Chang et al. 2017; Hikke et al. 2014; Karlsen et al. 2012; López‐Dóriga et al. 2001; Skjold et al. 2016; Thim et al. 2012; Thorarinsson et al. 2021; Thorarinsson, Ramstad, et al. 2024; Xu et al. 2012), these results may not directly translate to field performance due to varying environmental and biological factors (Karlsen et al. 2012). Evaluating vaccine effectiveness under relevant field conditions is therefore crucial to understanding their impact at the population level. For example, Jensen et al. (2012) analysed observational data from 198 commercial salmon cohorts and found that PD vaccination was associated with less outbreaks, mortality and discarded fish at slaughter. Karlsen et al. (2012) reported improved protection during a PD outbreak in a controlled field trial, focusing solely on survival outcomes. Moreover, Røsæg et al. (2021) evaluated both growth and mortality, demonstrating superior performance of the DNA vaccine Clynav compared to oil‐adjuvanted inactivated PD vaccines in terms of increased growth and reduced mortality.

Randomised controlled trials (RCTs) are considered the gold standard for determining causal relationships, as they minimise selection and confounding biases, although they may still be subject to other biases, such as misclassification. A cohabitant group design within the same rearing unit is robust, as study groups are exposed to identical conditions. In the field, however, conditions can vary substantially across space and time due to factors such as temperature, currents, fish handling, health status and the severity and dynamics of the target disease. Although observational studies can often evaluate effects under diverse conditions, scaling up the number of study units (e.g., sea cages) in controlled field trials is often impractical due to the resource‐intensive requirements of tagging and individual‐level data recording. Reproducing controlled field trials is therefore important to explore cage‐to‐cage variation and improve the generalizability of intervention effects under varying conditions.

This article presents the results of a controlled field trial conducted in a single sea cage at a commercially operated salmon farm. The aim of the trial was to evaluate the effects of the two licensed PD vaccines in Norway, ALPHA JECT micro 1 PD and Clynav, on growth and mortality during a PD outbreak.

## 2 Materials and Methods

### 2.1 Study Outline

The study was a controlled field trial with three groups, monitored from vaccination to harvest. These groups represented a subset of the total fish population within a single aquaculture unit (tank/sea cage) reared under commercial conditions. Vaccination of the study groups was performed on 23 January 2023. The fish were transferred to sea 79 days post‐vaccination, on 12 April 2023, and harvested 557 days post‐vaccination, on 2 August 2024.

The study population consisted of 46,046 Atlantic salmon ( *Salmo salar* ) of the Salmobreed strain, originating from a batch of 4.2 million eggs incubated on 30 June 2022. The fish were reared at a freshwater site (Site No. 13958, Follafoss, SalMar Settefisk AS) according to SalMar's standard procedures.

Immediately after vaccination, the three groups were mixed with 104,042 unmarked fish in the same tank, where they remained until sea transfer. At sea transfer, the tank was combined with 53,278 fish from another tank, containing fish from the same batch with a similar weight profile. A total of 176,675 fish were then transported by well boat to a sea cage with a 157‐m circumference at the designated sea site (Site No. 28636, Rataren). The fish were reared together in a single sea cage throughout the on‐growing phase until harvest. The sea site contained a total of seven cages during the production cycle.

### 2.2 Vaccination

The study fish were fasted for 4 days before vaccination. Tricaine methanesulphonate (Tricain, PHARMAQ) was used as an anaesthetic during the procedure. All vaccines were administered using an automatic vaccination machine (Maskon, Stjørdal, Norway) following standard protocols. Fish weighing less than 35 g were excluded from vaccination.

The study groups were vaccinated sequentially, with each group processed immediately after the previous one. This took place midway through the vaccination of the entire tank population and was completed within a single day. After passing through the vaccination machine, the study groups were marked for identification by clipping either the adipose fin alone or in combination with the left or right maxilla. One group received AJm1PD, another received Clynav, and the third remained unvaccinated against PD, serving as a negative control. In addition to their respective PD vaccine assignments, all study groups received a hexavalent oil‐based core vaccine (AJm6) and a monovalent water‐based vaccine (AERM), as detailed in Tables 1 and 2. The unmarked fish cohabiting with the study groups throughout the study received AJm6, AERM and Clynav.

**TABLE 1:** Overview of the vaccines administered to the study population.

Vaccine name	ALPHA JECT micro 6^a^	Alpha ERM Salar^b^	Clynav^c^	ALPHA JECT micro 1 PD^d^
Abbreviation	AJm6	AERM	Clynav	AJm1PD
Dose volume (mL)	0.05	0.025	0.05	0.05
Administration route	Intraperitoneal (IP)	Intraperitoneal (IP)	Intramuscular (IM)	Intraperitoneal (IP)
Recommended immunisation period (day degrees)	520 (600 for IPNV)	520	399	516
Marketing authorization holder (MAH)	PHARMAQ part of Zoetis	PHARMAQ part of Zoetis	MSD Animal Health	PHARMAQ part of Zoetis

^a^ ALPHA JECT micro 6 is a hexavalent oil‐adjuvanted vaccine containing formaldehyde‐inactivated *Aeromonas salmonicida* subsp. *salmonicida*, *Aliivibrio salmonicida* , *Listonella anguillarum* serotype O1, *L. anguillarum* serotype O2a, *Moritella viscosa* and infectious pancreatic necrosis virus (IPNV). For more details, see SPC: https://medicines.health.europa.eu/veterinary/en/600000014282.

^b^ Alpha ERM Salar is a monovalent water‐based vaccine containing formaldehyde‐inactivated *Yersinia ruckeri* serotype O1b. For more details, see SPC (in Norwegian only): https://medicines.health.europa.eu/veterinary/en/600000038828.

^c^ Clynav is a monovalent vaccine containing DNA plasmids encoding for SAV proteins. For more details, see SPC: https://www.ema.europa.eu/en/documents/product‐information/clynav‐epar‐product‐information_en.pdf.

^d^ ALPHA JECT micro 1 PD is a monovalent vaccine containing formaldehyde‐inactivated SAV. For more details, see SPC: https://medicines.health.europa.eu/veterinary/en/documents/download/687eca50‐772a‐4ad8‐8066‐94e66c566aa2.

**TABLE 2:** Overview of the study groups with vaccines, marking and estimated number of vaccinated fish.

Group	Vaccines	Marking^a^	Number of fish^b^
AJm1PD	AJm6 + AERM + AJm1PD	RM + AF	15,502
Clynav	AJm6 + AERM + Clynav	LM + AF	15,100
Negative control	AJm6 + AERM	AF	15,444

^a^ AF = adipose fin; LM = left maxilla; RM = right maxilla.

^b^ Number of doses estimated by the automatic vaccination machine (Maskon, Stjørdal, Norway).

The water temperature during vaccination was 11°C, while temperatures between vaccination and sea transfer ranged from 7.4°C to 12.2°C. The immunisation period from vaccination to sea transfer was estimated at approximately 755° days, well within the recommended range for all vaccines (Table 1).

### 2.3 Monitoring and Data Collection

Regulations required monthly visits by fish health professionals to assess health and conduct diagnostics. During the sea phase, mandatory monthly sampling of heart tissue from moribund or fresh dead fish were conducted for SAV PCR analysis. In addition, heart tissue from 30 to 32 randomly selected fish per group was collected at the harvest line for SAV PCR analysis.

Weights were recorded at several points throughout the study. At vaccination, individual weights were measured for 51–58 fish per group. At harvest, 489–500 fish per group were weighed. Weights were also recorded for all fish assessed for abdominal local reactions.

Daily mortality data were collected by the production site staff, with group markings recorded. The staff remained blinded to group identities. At the sea site, mortalities were also categorised by cause, following the methodology outlined by Aunsmo, Bruheim, et al. (2008).

Vaccine‐induced local reactions, including abdominal adhesions and melanisation, were assessed in 10–29 fish per group at 35 and 64 weeks of post‐vaccination. Abdominal adhesions were scored following the methodology described by Midtlyng et al. (1996), and a total adhesion score (0–6), derived from the highest regional score, was used as the output variable. Any fish assigned a total adhesion score of 0 (indicating no abdominal adhesions) were considered unvaccinated and excluded from the dataset. Melanin deposits on the viscera and abdominal wall were evaluated and scored 0–3 according to standard industry guideline.

An increased risk of spinal deformities, described as cross‐stitch vertebrae, has been associated with the use of inactivated SAV vaccines, although a direct causal relationship has not been established (Thorarinsson, Negaard, et al. 2024; Trangerud et al. 2020). Severe occurrences of such lesions can lead to reduced fish growth (Thorarinsson, Negaard, et al. 2024). To monitor this, 99 fish per group were sampled at the harvest line and assessed for cross‐stitch vertebrae using X‐ray imaging. Each fish was scored on a scale from 0 to 3, where 0 indicated no visible damage and 3 indicated multiple affected areas.

Sampling for data collection was carried out randomly and progressively throughout both the vaccination and harvest processes to ensure a representative selection. At the sea site, however, progressive sampling was impractical, so fish were collected randomly using a seine net.

### 2.4 Data Management and Analysis

Weight data were analysed using a one‐way ANOVA to compare group means. Pairwise comparisons were performed using Tukey's HSD test to identify differences between groups. The residuals were inspected to evaluate model assumptions of normality and homoscedasticity.

Cause‐specific classification of mortalities is conducted at the cage level rather than the individual level, making it unsuitable for evaluating PD‐related deaths between groups. Therefore, total mortality, based on group markings during the defined PD outbreak period, was analysed to assess differences between groups. A chi‐square test of independence was performed to evaluate whether group membership significantly affected mortality. Pairwise comparisons of mortality between groups were conducted using chi‐square tests without continuity correction, with *p*‐values adjusted for multiple testing using Holm–Sidak's method.

Manual inspection of mortalities by farm staff to determine group identity can be prone to misclassification (Røsæg et al. 2021). In the current study, misclassification may occur on two levels: between maxilla‐clipped and adipose fin‐clipped fish, and between marked and unmarked fish. To address this, the two PD vaccine groups were deliberately assigned distinct maxilla clips (right or left) to enhance comparability between the two test groups. Moreover, bias‐adjusted effect sizes were calculated to assess the direction and magnitude of potential misclassification bias (Dohoo 2014).

Abdominal local reaction scores from fish sampled during the sea phase of production, along with Ct‐values from heart tissue samples and spinal deformity scores from fish collected at the harvest line, were analysed using the Kruskal–Wallis test to assess differences among groups. For abdominal local reaction scores, analyses were conducted separately for each score type (total adhesions, melanin deposits on the viscera and melanin deposits on the abdominal wall) and for each sampling point. PCR‐negative samples were assigned the assay's cutoff Ct value (33.43) to ensure their consistent inclusion in the analysis. If the Kruskal–Wallis test indicated significant differences, pairwise comparisons were performed using Dunn's test with adjustment for multiple comparisons.

All statistical analyses were conducted using GraphPad Prism (version 10.4.1), with the significance level defined as *p* < 0.05.

### 2.5 Ethics Statement

The study fish were reared and monitored in accordance with Norwegian legislation and fish owner's SOPs. Clipping of the adipose fin and maxilla are established methods for marking salmon, ensuring effective group identification throughout the trial period with minimal impact on animal welfare. Both the marking procedure and the trial were approved by the Norwegian Food Safety Authority under FOTS approval number 29958.

## 3 Results

### 3.1 PD Diagnostics

**FIGURE 1:**
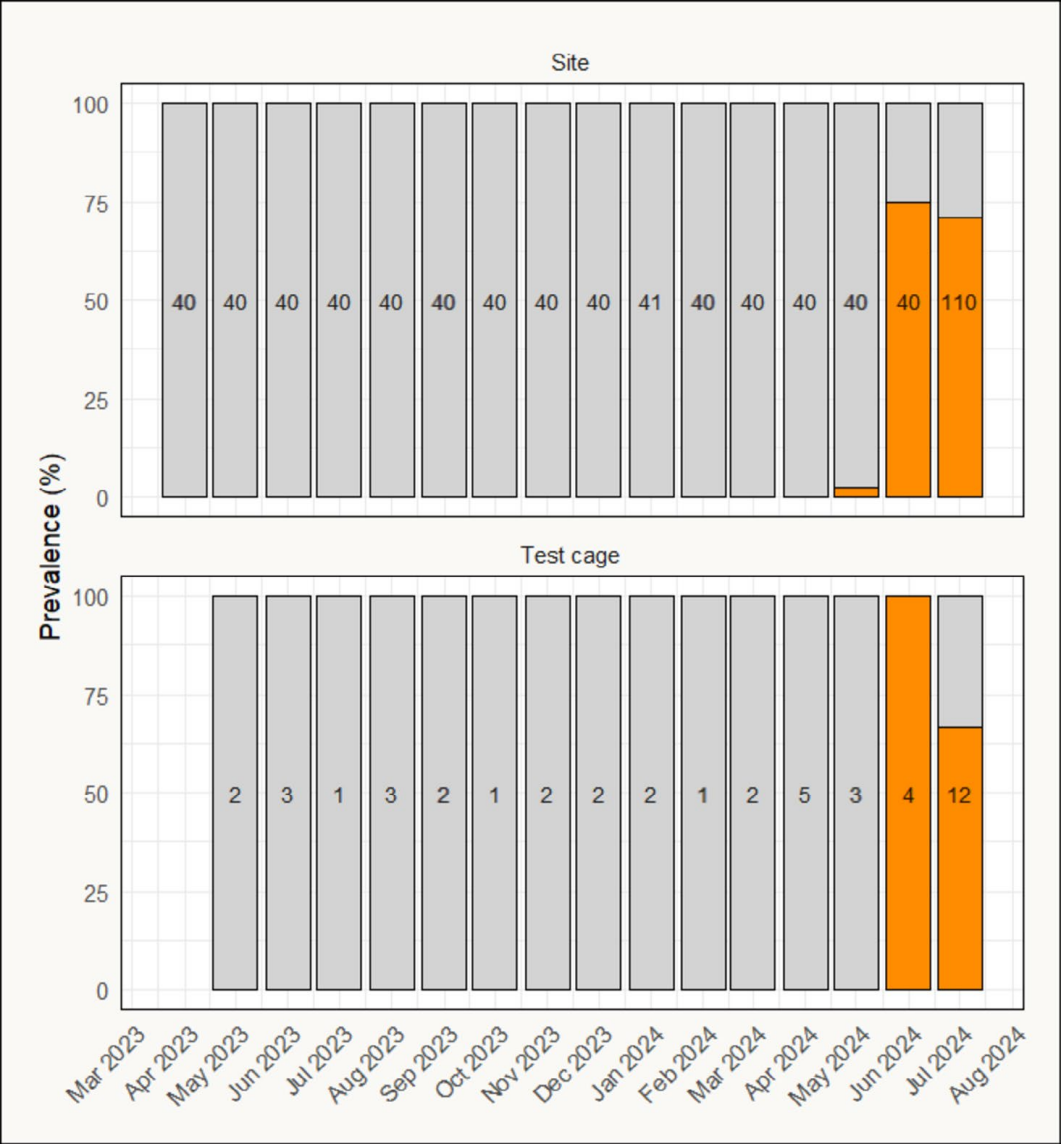
Monthly screening for SAV at the sea site with number of samples and prevalence.

SAV was first detected in 1 of 40 sampled fish during the monthly screening at the sea site on 11 May 2024, 13 months after sea transfer. Consequently, the site was reported suspicious of PD to the authorities, in accordance with the regulations. The virus was identified as SAV2 by specific PCR. In the following 2 months, prevalence increased to 75% in June and 80% in July (Figure 1). In the test cage, SAV2 was first detected in four sampled fish on 12 June 2024, with Ct‐values below 21 in three of the four fish, indicating high viral loads. Based on these findings, the disease outbreak period for the test cage was defined as June 1 to the final harvest day on August 2. However, a PD diagnosis was formally confirmed for the site on June 14, following the collection of formalin‐fixed tissue samples and histological examinations.

**FIGURE 2:**
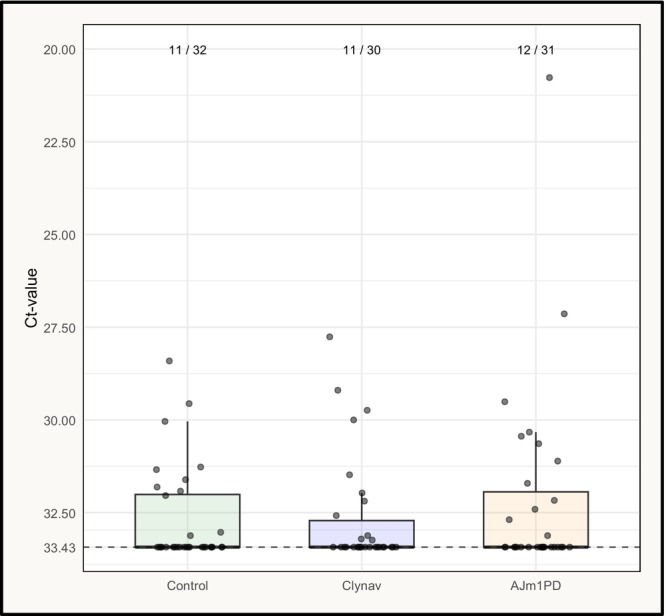
SAV PCR data from heart tissue samples collected at the harvest line. The horizontal dashed line indicates the Ct‐value cutoff level for the SAV PCR assay. The ratio above the boxes indicates the prevalence.

SAV PCR analysis of harvest line samples revealed a detection prevalence ranging from 34% to 39% across the groups, with overall Ct values in the higher range, which may indicate that the viraemic phase occurred before harvest (Figure 2). The Kruskal–Wallis test showed no significant differences in Ct values among the groups (*p* = 0.876); therefore, no pairwise comparisons were performed.

### 3.2 Mortality

During the vaccination of the study groups, a technical malfunction caused a small group of fish (~150–200 fish) to remain on the vaccination table for an extended period. This likely contributed to the elevated mortality observed the following day in the Clynav group, with 125 dead fish compared to 25 and 8 dead fish in the control and AJm1PD groups, respectively.

Twenty days post‐vaccination, an outbreak of heart and skeletal muscle inflammation (HSMI) was detected, lasting approximately 1 week. This outbreak resulted in high mortality levels, making it infeasible for staff to count all mortalities based on group marking. During the HSMI outbreak period, a minimum of 100 dead fish were instead categorised daily according to their markings, and this information, combined with the counted total daily mortality in the tank, was used to estimate mortality per study group.

At the hatchery, there were no mortality differences between the PD‐vaccinated groups (*p* = 0.98), while mortality was significantly higher in the adipose‐fin‐clipped group (control group) compared to the maxilla‐tagged groups (PD‐vaccinated groups) (*p* < 0.0001) (Table 3). Given that PD does not occur in freshwater and the vaccines are expected to exhibit similar safety profiles, this mortality disparity—along with the technical failure affecting the Clynav group (maxilla‐tagged)—may suggest misclassification.

**TABLE 3:** Overview of the estimated number of fish and mortalities in the fish population from vaccination until harvest.

		Control	AJm1PD	Clynav	Unmarked	Test tank/cage
Freshwater phase	Vaccinated	15,444	15,502	15,100	104,042	150,088
	Mortalities	3290 (21.3%)	2618 (16.9%)	2552 (16.9%)	18,231 (17.5%)	26,691 (17.8%)
	Added fish				53,278	
Seawater phase	Sea transferred	12,154	12,884	12,548	139,089	176,675
	Pre‐outbreak mortalities	401 (3.3%)	345 (2.7%)	348 (2.8%)	9112 (6.6%)	10,206 (5.8%)
	Post‐outbreak mortalities	292 (2.5%)	236 (1.9%)	169 (1.4%)	4689 (3.6%)	5386 (3.2%)
	Total mortality seawater phase	693 (5.7%)	581 (4.5%)	517 (4.1%)	13,801 (9.9%)	15,592 (8.8%)

**FIGURE 3:**
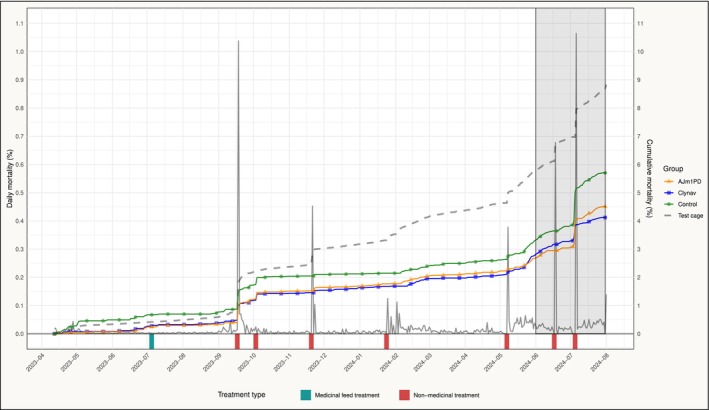
Daily and cumulative mortality (%) in the test cage (grey) and cumulative mortality (%) for the study groups during the seawater phase of production, with lice treatments depicted. The shaded area represents the defined PD outbreak period.

In the seawater phase, mortality was primarily associated with non‐medicinal delousing treatments, which led to acute mortality and wound development (Figure 3). The test cage was deloused once using a feed treatment (emamectin benzoate) and seven times using Hydrolicer (produced by Smir AS) or a combination of Hydrolicer and freshwater. Additionally, cardiomyopathy syndrome (CMS) was detected in June 2024. In the period leading up to slaughter, a proportion of dead fish was classified as CMS and PD mortalities by site personnel, indicating a concurrent outbreak of both diseases.

**FIGURE 4:**
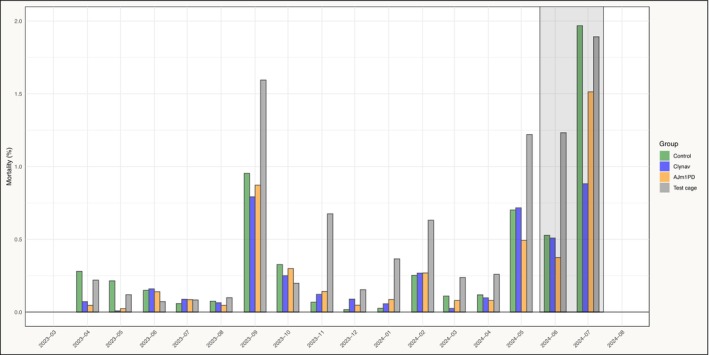
Monthly mortality figures (%) in the test cage (grey) and the study groups during the seawater phase of production. The shaded area represents the defined PD outbreak period.

A significant difference in mortality was observed between the groups during the seawater phase before the PD outbreak (*p* = 0.0074). Pairwise comparisons showed that both PD‐vaccinated groups differed significantly from the control group, while no difference was found between the two PD vaccine groups (*p* = 0.64) (Table 4). To investigate potential misclassification between the groups, the two PD‐vaccinated groups (both maxilla‐clipped) were pooled and compared to the control group (adipose fin‐clipped). This analysis revealed that the mortality differences were primarily linked to the first 2 months after sea transfer (*p* < 0.0001). Beyond this initial period, mortality patterns appeared random, with no significant differences between the groups until the PD outbreak (*p* = 0.35). This suggests that misclassification between groups was not systematic throughout the entire production cycle. In contrast, a systematic and substantial difference was observed between marked and unmarked fish from the first lice treatment and throughout the seawater phase (Figures 3 and 4).

**TABLE 4:** Summary of mortality differences (95% CI), risk ratios (95% CI) and *p*‐values (chi‐square test) before and during the PD outbreak, including misclassification‐adjusted estimates for the outbreak period (*p*‐values are adjusted for multiple comparisons using Holm–Sidak's method).

Pairwise comparisons	Before PD outbreak			During PD outbreak					
	(12 April 2023–31 May 2024)			(1 June 2024–2 August 2024)					
	Unadjusted estimates			Unadjusted estimates			Misclassification adjusted estimates		
	RD (95% CI)	*p*	RR (95% CI)	RD (95% CI)	*p*	RR (95% CI)	RD (95% CI)	*p*	RR (95% CI)
AJm1PD: control	−0.62 (−1.05 to −0.19)	0.011	0.81 (0.70–0.94)	−0.60 (−0.98 to −0.22)	0.0026	0.76 (0.64–0.90)	−0.99 (−1.53 to −0.45)	0.00026	0.81 (0.72–0.90)
Clynav: control	−0.53 (−0.96 to −0.09)	0.032	0.84 (0.73–0.97)	−1.10 (−1.45 to −0.74)	< 0.0001	0.56 (0.46–0.67)	−2.07 (−2.58 to −1.56)	< 0.0001	0.59 (0.52–0.67)
Clynav: AJm1PD	0.10 (−0.31–0.50)	0.64	1.04 (0.89–1.20)	−0.50 (−0.82 to −0.17)	0.0026	0.74 (0.61–0.90)	−1.08 (−1.56 to −0.60)	< 0.0001	0.74 (0.64–0.84)

Two lice treatments were conducted during the PD period, with the second treatment resulting in the highest treatment‐related mortality observed during production (Figure 3). Before the first delousing, the Clynav group exhibited the highest average daily mortality rate (0.026%), followed by the control group (0.022%) and the AJm1PD group (0.017%). In the period between the two treatments, the control group had the highest rate (0.019%), with lower rates observed in the Clynav group (0.014%) and the AJm1PD group (0.012%). After the second treatment, more pronounced differences emerged, with a shift in mortality patterns between the PD‐vaccinated groups: the control group achieved the highest average daily mortality rate (0.064%), followed by the AJm1PD group (0.050%) and the Clynav group (0.026%).

Over the PD outbreak period, mortality differed significantly between groups (*p* < 0.0001). Pairwise comparisons showed that the Clynav group displayed significantly lower mortality compared to both the AJm1PD group (*p* = 0.0026) and the control group (*p* < 0.0001). Similarly, the AJm1PD group had lower mortality compared to the control group (*p* = 0.0026). Bias‐adjusted risk differences were calculated to account for potential misclassification.^1^ The adjusted risk differences were higher than the unadjusted values (Table 4), suggesting that ignoring misclassification biases the results toward the null, thus underestimating the true effect size. Risk ratios, in contrast, were largely unaffected by these adjustments.

### 3.3 Weight

No significant differences in weights were observed between the groups at the time of vaccination (ANOVA, *p* = 0.27). The mean weights, along with their 95% confidence intervals, were as follows: control (60.6 g, 95% CI: 58.4–62.8 g), AJm1PD (59.0 g, 95% CI: 57.0–61.0 g) and Clynav (58.2 g, 95% CI: 56.1–60.3 g). These findings suggest that any subsequent differences in slaughter weight are unlikely to be due to initial weight disparities.

**FIGURE 5:**
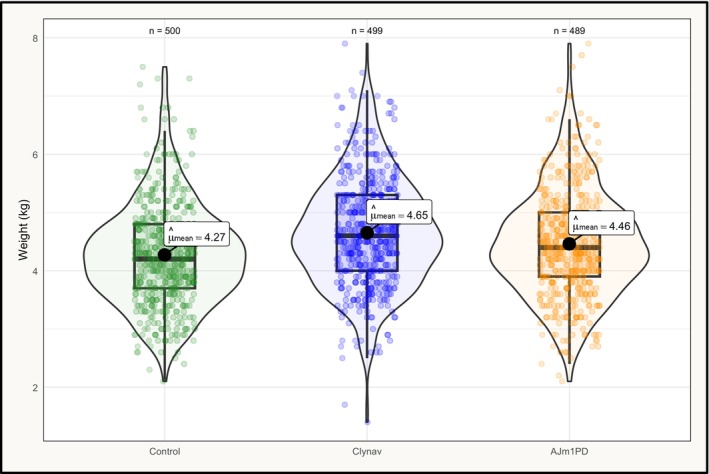
Violin and box plots of weight measurements from fish sampled at the harvest line.

Mean harvest weights (kg) were measured as: control (4.27, 95% CI: 4.20–4.35), AJm1PD (4.46, 95% CI: 4.38–4.55) Clynav (4.65, 95% CI: 4.57–4.73). A one‐way ANOVA revealed a significant effect of group on weights (*p* < 0.0001). Tukey's pairwise comparisons demonstrated that both Clynav and AJm1PD had significantly higher weights than control, with Clynav also having significantly higher weights than AJm1PD (Table 5 and Figure 5).

**TABLE 5:** Pairwise comparisons of harvest weights (kg) with 95% confidence intervals (CI) and *p*‐values from Tukey's HSD test.

Group comparisons	Mean difference (95% CI)	*p*
AJm1PD: control	0.19 (0.05–0.33)	0.0031
Clynav: control	0.38 (0.24–0.51)	< 0.0001
Clynav: AJm1PD	0.19 (0.05–0.32)	0.0036

### 3.4 Local Reactions

**FIGURE 6:**
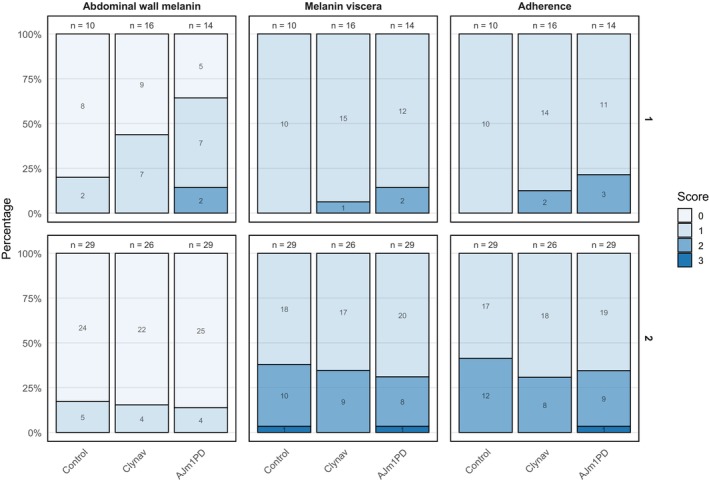
Frequency plot over local reaction scores at first sampling 6 months post‐vaccination (upper row) and second sampling 12 months post‐vaccination (lower row).

No significant differences in local reaction scores were observed between the treatment groups across the sampling points, 6 and 12 months post‐vaccination (Figure 6). Statistical analysis using the Kruskal–Wallis test revealed no evidence of group differences for adherence scores (*p* = 0.30 at the first sampling and *p* = 0.72 at the second sampling), melanin in the viscera (*p* = 0.42 and *p* = 0.86, respectively), or melanin in the abdominal wall (*p* = 0.069 and *p* = 0.94, respectively). While there was an indication that AJm1PD had slightly higher scores for abdominal wall melanin at the first sampling (*p* = 0.067), this trend was weak and not consistent across sampling points. Overall, all categories were well within the normal range as defined in the summary of product characteristics (SPC) for the applied vaccines.

### 3.5 Spinal Deformities

X‐Ray examination of 99 fish per group revealed a very low overall prevalence of spinal deformities, described as cross‐stitch vertebrae (Table 6). Minimal variation was observed between groups, with no statistically significant differences detected (Kruskal–Wallis, *p* = 0.90).

**TABLE 6:** Frequency of cross‐stitch vertebrae scores (0–3) assessed by X‐ray imaging of fish sampled at the harvest line. Data represent the number of fish assigned to each score.

Group	*n*	Cross‐stitch vertebrae			
		0	1	2	3
Control	99	95	3	0	1
Clynav	99	96	2	0	1
AJm1PD	99	95	3	1	0

## 4 Discussion

The objective of this study was to evaluate the effects of Clynav and AJm1PD on growth and mortality during a PD outbreak. Clinical PD induced by SAV2 was confirmed in June 2024, 14 months after sea transfer. Both vaccines were associated with significantly better growth and lower mortality compared to the control group, while Clynav demonstrated significantly lower mortality and higher growth rates than AJm1PD.

The current study shares many similarities with that of Røsæg et al. (2021), and the results for Clynav are consistent with their findings. Their study reported a significant reduction in mortality of 1.31% (CI: 0.8–1.8) in a cage that included only Clynav and a control group. In contrast, a separate cage with AJm1PD and a control group showed no differences. Due to potential differences in disease dynamics and progression between the two cages, it was difficult to draw definitive conclusions about the effect of AJm1PD on mortality relative to Clynav. However, while the PIT‐tag component of the Røsæg et al. (2021) study—which included one sea cage with several cohabitant groups—found no significant difference in mortality for either Clynav or AJm1PD compared to the control, the relative ranking of the vaccines aligns with the current study. Røsæg et al. (2021) also reported that Clynav improved harvest weight by 0.43 kg (CI: 0.29–0.57) and 0.51 kg (CI: 0.36–0.65) in two separate sea cages. In contrast to our findings, they observed no effect of AJm1PD on growth. The reason for this discrepancy remains unclear. Both studies were conducted in the same geographic region using spring smolt exposed to similar temperature profiles. Local reaction scores were considered normal in both studies. Neither study observed cross‐stitch vertebrae, a condition linked to growth impairment (Thorarinsson, Negaard, et al. 2024). Key differences between the studies include fewer delousing treatments, the use of a different smolt strain, the unmarked group being unvaccinated against PD, which may have influenced infection pressure in the cage, and the presence of both SAV2 and SAV3 in Røsæg et al. (2021), whereas only SAV2 was detected in our study. In Røsæg et al. (2021), SAV was also detected earlier, and a longer interval elapsed between the clinical outbreak and slaughter. This factor may have influenced the observed effect sizes, as longer‐term consequences were likely not fully captured in the present study. For example, it may help explain the larger harvest weight effects reported for Clynav in their study. Our study also experienced a concurrent CMS outbreak, and systemic immune activation and potential viral interactions could, in theory, affect vaccine responses.

We found that the groups vaccinated with PD vaccines had significantly higher harvest weights compared to the control group: 0.19 kg (95% CI: 0.05–0.33) for AJm1PD and 0.38 kg (95% CI: 0.24–0.51) for Clynav. These differences indirectly support previous findings that PD caused by SAV2 can lead to substantial growth loss (Røsæg, Garseth, et al. 2019), although this cannot be directly quantified in our study, as we lacked an uninfected control group for comparison. Our findings could have been strengthened by more robust weight measurements before disease onset. While weights were recorded from 26 to 29 fish per group during the April 2024 sampling, approximately 1 month before SAV detection, no statistical differences were found (ANOVA *p* = 0.41; AJm1PD: 3711 g [95% CI: 3413–4008], Clynav: 3781 g [3526–4036], control: 3526 g [3223–3828]). However, the small sample size and limited representativeness—due to manual surface collection—limit the strength of these data, and they were therefore not included in the results. Without representative pre‐outbreak measurements, some uncertainty remains as to whether the observed weight differences between AJm1PD and Clynav were solely due to differences in PD protection. For instance, the larger intraperitoneal dose in the AJm1PD group may have entailed a higher biological cost, despite similar local reaction scores. It is known that oil‐based vaccines can reduce growth, and that this effect may occur independently of local reaction scores (Aunsmo, Larssen, et al. 2008). Although detailed on‐site weight measurements are currently challenging, advancements in camera technology may enable automated detection of identification marks, allowing for more precise tracking of when weight discrepancies arise during production (Fari and Skjåvik 2024).

The HSMI outbreak was a major driver of the higher mortality levels during the freshwater phase compared to the seawater phase (Table 3). Since none of the vaccines in the study contain a component against HSMI, we do not expect any of the groups to have been favoured by this outbreak. The accumulated mortality in the test cage during the sea phase ended at 8.8%, which is below the national average cumulative mortality risk per calendar year of 16.7% for the same period (Sommerset et al. 2024). Mortality was primarily associated with lice treatments, but an increase in mortality was also observed between treatments during the final production months. Two lice treatments were conducted during the PD period, with the last treatment resulting in high treatment‐related mortality (Figure 3). The observed shift in mortality between the study groups following these treatments may be attributed to two mechanisms: handling stress of infected fish may have triggered the development of clinical PD (Jansen et al. 2017), while diseased and weakened fish may have been more prone to mortality (Overton et al. 2019). It is possible that the Clynav group experienced the mildest disease progression after the first treatment, allowing them to tolerate the second treatment better than the other two groups. The underlying cause of the higher mortality observed in the Clynav group during the outbreak phase before these two treatments remains unclear (Figure 3).

The study highlights certain methodological considerations for mortality as a vaccine endpoint. First, defining a relevant disease period is difficult, and this can affect the effect size estimate. An overly long period risks diluting vaccine effects by including mortality unrelated to PD. Conversely, a period that is too short may overestimate effects by focusing solely on acute mortality or underestimate effects by excluding delayed mortalities, such as chronic disease progression. Second, the choice of effect sizes can influence interpretation. Risk differences (RD) is less sensitive to background mortality and provide a more stable measure of absolute risk reduction. In contrast, risk ratio (RR) can be misleading when overall mortality is very high or very low, as it may exaggerate or dilute effect sizes. Given the presence of concurrent diseases and mortality events, RD likely offers a more reliable estimate of vaccine effects in this study. Furthermore, the mortality rate in the study groups would be expected to align with that of the test cage during the pre‐outbreak period; however, a notable discrepancy was observed throughout the study, with increasing discrepancies between marked and unmarked fish correlating with acute mortality. Some of this may stem from the lack of randomization during vaccination or the inclusion of additional fish from a separate tank at sea transfer, despite these fish were part of the same fish group with comparable size and history. A more likely factor, however, is the challenge faced by site personnel in identifying marked fish from unmarked fish, particularly during high mortality events. In contrast, no clear link was observed between acute mortality and suggested misclassification between the adipose‐ and maxilla‐clipped groups during the pre‐outbreak sea phase. A noticeable skewness occurred during the first 2 months after sea transfer, but thereafter, the mortality patterns of the groups appeared similar. This may reflect a learning curve among sea site personnel or delays in communication about the marking system. Additionally, except for the initial period after transfer, it seems that when site staff examined dead fish, both adipose fin and maxilla were consistently inspected. Misclassification could potentially have been reduced through training and predefined guidelines for recording tagged mortalities, particularly during periods of high acute mortality (e.g., by limiting tag reading to a subset of 100 fish, as was done at the hatchery during the HSMI outbreak).

Adjusting for misclassification can provide insight into the bias direction and strength but may require certain assumptions (Dohoo 2014). We assumed equal pre‐outbreak mortality risk across categories (adipose vs. maxilla and marked vs. unmarked), based on the established safety profile of these vaccines over several years of use in production. Moreover, our bias adjustment during the outbreak assumed a proportional distribution of mortality across study groups. This increased the overall observed mortality, inflating RD, while the RR remained unchanged. Adjustments for misclassification during the pre‐outbreak phase aimed to correct group sizes entering the outbreak phase but had limited impact, as group sizes did not change by large numbers. The differences in RR after adjustment reflected the revised group sizes (see Table 4 and Supporting Information S1). It would have been possible to adjust for potential misclassification from maxilla‐clipped groups to the adipose fin‐clipped group during the outbreak phase, using observed misclassification rates from the pre‐outbreak phase. However, this was not pursued, as there was no convincing evidence of systematic misclassification throughout production, making it a poor predictor for the outbreak phase. Such an adjustment would have had minimal impact on effect sizes when comparing the two maxilla‐clipped groups but would have shifted RD and RR toward null in comparisons against the adipose fin‐clipped control group. This illustrates the complexity of the study design, where different types of misclassification biases can skew results in opposing directions. These considerations highlight the importance of evaluating how fish identification methods may affect outcomes such as mortality when planning experimental design. An alternative method for assessing mortality could involve estimating survival based on recapture probabilities through structured sampling at the harvest line (Røsæg et al. 2021). Misclassification bias is less likely to affect weight measurements, as these were performed by dedicated personnel under controlled conditions at onshore facilities.

A strength of the study is that all experimental groups were reared in the same production unit throughout the trial. However, a potential bias may arise if a group within the population exhibits altered behaviour (e.g., diseased fish aggregating near the surface) (Røsæg, Rimstad, et al. 2019). This can be problematic for at least two reasons. First, it makes the results more vulnerable to events associated with stressful handling, such as delousing treatments, which are common in modern salmon farming. For instance, a temporary technical failure leading to mortality during a delousing treatment could introduce significant bias if the groups are not well mixed within the population. Second, it may lead to selection bias, as samples could be drawn from different distributions within each group (e.g., selectively sampling healthier individuals within a diseased group). The latter issue can to some extent be mitigated by counting the number of fish required to achieve the predefined sample size within each group and estimating sampling probabilities (Røsæg et al. 2021). One limitation of this study is that such a procedure was not implemented.

Another limitation of the study design is the absence of complete randomization of the groups during vaccination. The study fish originated from the same cohort and had undergone identical handling up to the point of vaccination. The groups were vaccinated sequentially; however, weight samples were taken throughout the vaccination process to ensure a representative sample. These measurements demonstrated a consistent weight distribution across the groups, reducing the likelihood that the lack of randomization introduced substantial bias.

## 5 Conclusion

This controlled field trial supports the effectiveness of current PD vaccines for Atlantic salmon, largely consistent with previous studies. Vaccinated groups exhibited significantly improved growth and reduced mortality compared to the control group. However, the DNA‐based vaccine Clynav showed significantly greater effect sizes than the inactivated virus vaccine, ALPHA JECT micro 1 PD. The study also highlights the potential impact of misclassification bias on mortality outcomes in a field setting, where bias could influence results in either direction. In this study, failure to account for misclassification is suggested to have led to an underestimation of vaccine effects (risk difference).

## Author Contributions

The authors take full responsibility for this article.

## Conflicts of Interest

Jostein Mulder Pettersen is employed by Salmalytics and was contracted by PHARMAQ part of Zoetis to participate in this study. Marius Karlsen, Marte Follesø Sønnervik, Petter Gjesdal, Hege Jørstad Sekkenes and Ane Sandtrø are employed by PHARMAQ part of Zoetis. PHARMAQ part of Zoetis, is the manufacturer of ALPHA JECT micro 1 PD. Geir Schriwer and Magnus Vikan Røsæg are employed by SalMar, which owns the fish included in this study.

## Supporting information


Data S1.

## Data Availability

The data that support the findings of this study are available from the corresponding author upon reasonable request.

## Endnotes

^1^
The quantitative bias analysis accounted for potential misclassification through targeted adjustments. To correct group sizes entering the outbreak period, adjustments were made for suggested misclassification between adipose fin‐ and maxilla‐clipped groups and between marked and unmarked fish from vaccination until the PD outbreak, under the assumption of equal mortality risk among these categories during the adjustment process. During the outbreak period, adjustments were made between Clynav (marked) and unmarked fish, as these were the only two groups that had received identical vaccines. These misclassified fish were redistributed based on group‐specific mortality rates. No adjustments were made between adipose fin‐ and maxilla‐clipped groups during the outbreak, nor between marked and unmarked fish in the hatchery phase, as there was no basis to suggest misclassification in these contexts. See Supporting Information S1 for detailed methodology.
